# Mixed-strain infection and heteroresistance in drug-resistant tuberculosis: insights from a whole-genome sequencing pilot

**DOI:** 10.3389/fepid.2026.1813571

**Published:** 2026-08-06

**Authors:** Mandlenkosi Manika, Lindiwe Modest Faye, Ntandazo Dlatu, Mojisola Clara Hosu, Teke Apalata

**Affiliations:** 1 Tuberculosis and Associated Research and Innovation Platform (TARIP), School of Pathology, Faculty of Medicine and Health Sciences, Walter Sisulu University, Mthatha, Eastern Cape, South Africa; 2 Walter Sisulu Institute for Clinical Governance, Healthcare Administration, School of Public Health, Faculty of Medicine and Health Sciences, Walter Sisulu University, Mthatha, South Africa

**Keywords:** DR-TB, epidemiology, hetororesistance, mixed strain, tuberculosis, whole genome sequencing

## Abstract

**Background:**

Mixed-strain *Mycobacterium tuberculosis* (*M.* tuberculosis; Mtb) infections and heteroresistance are increasingly recognized as under-detected contributors to drug resistance dynamics. In high-burden rural settings such as the Eastern Cape, these genomic features may reflect both within-host diversity and ongoing transmission. However, their epidemiological and clinical implications remain incompletely understood, particularly in contexts with high HIV co-infection.

**Methods:**

We conducted an exploratory whole-genome sequencing (WGS) analysis of 28 drug-resistant *M.* tuberculosis isolates obtained through routine diagnostic workflows. Mixed-strain candidacy was defined using a composite genomic criterion, including multi-lineage assignments, ≥2 heteroallelic variants [allele frequency (AF): 0.10–0.90], or heteroresistance across multiple drug classes. A Random Forest (RF) model with Leave-One-Out Cross-Validation (LOOCV) was used as a feature-prioritization tool to identify genomic characteristics associated with mixed-strain candidacy. Given the limited sample size, analyses were designed to generate hypotheses rather than support causal inference. Clinical and epidemiological metadata, including HIV status and treatment outcomes, were not available.

**Results:**

Lineage 4 (46%) and Lineage 2 (43%) predominated, with 10.7% of isolates classified as probable mixed-strain candidates. Heteroallelic variants were most frequently observed in *fabG1/inhA* and *rpoB*, with a median AF of 0.22, consistent with subclonal diversity and possible within-host microevolution. Additional heteroallelic variants were identified in non-resistance-associated genes, suggesting broader genomic heterogeneity. The RF model demonstrated high discriminatory performance (AUC = 1.00), although this likely reflects the small sample size and should be interpreted cautiously. Feature importance analysis identified heteroresistance burden as the most prominent predictor of mixed-strain candidacy.

**Conclusion:**

This pilot study provides preliminary genomic evidence of mixed-strain infection and heteroresistance in a high-burden rural setting. While these findings highlight the potential role of subclonal diversity in shaping resistance patterns, their clinical and epidemiological relevance remains uncertain due to the absence of patient-level data and limited sample size. Future studies integrating genomic, clinical, and epidemiological data in larger cohorts and incorporating benchmarking against established tools are required to validate these findings and clarify their implications for tuberculosis control.

## 1 Introduction

Drug-resistant tuberculosis (DR-TB) remains a major barrier to global TB elimination efforts, driven in part by undetected transmission and substantial within-host genetic diversity (1, 2). Two interrelated phenomena, mixed-strain infection (simultaneous infection with two or more distinct *Mycobacterium tuberculosis* lineages) and heteroresistance (the coexistence of drug-susceptible and drug-resistant subpopulations within a host), pose significant diagnostic and epidemiological challenges. These complexities can obscure true resistance profiles, potentially leading to suboptimal treatment regimens and misinterpretation of transmission dynamics (3, 4). In high-burden settings such as Botswana and South Africa, including the Eastern Cape province, where over 3,000 MDR/RR-TB cases have been reported in recent surveillance cycles, mixed infections have been associated with adverse treatment outcomes and increased mortality. However, the underlying mechanisms remain incompletely understood (4, 5). Conventional diagnostic approaches, particularly culture-based methods, may introduce biological and analytical biases. The prolonged culture process can favor the expansion of dominant clones, thereby masking minority variants and reducing the detectability of within-host diversity. Similarly, single-colony sequencing approaches may not capture the full spectrum of genomic heterogeneity present in clinical samples. Whole-genome sequencing (WGS) has emerged as a powerful tool to overcome these limitations by enabling high-resolution detection of minor resistance-associated variants and complex strain mixtures (6, 7). In addition to informing individualized treatment strategies, WGS supports molecular epidemiology by improving the resolution of transmission networks and identifying previously unrecognized transmission clusters (8). Advanced bioinformatic tools, including QuantTB and MixInfect2, have been developed to distinguish mixed infections from within-host microevolution using probabilistic and reference-based frameworks. However, these approaches have been primarily validated in well-resourced settings, and their applicability in rural, resource-limited contexts remains underexplored (9–11). Furthermore, genomic analyses are often conducted in isolation from clinical and epidemiological data, limiting their translational value for patient management and public health decision-making. This limitation is particularly relevant in high HIV prevalence settings, where host immune status may influence susceptibility to mixed infection, within-host evolution, and treatment response, yet remains underrepresented in genomic analyses. The clinical implications of mixed infections and heteroresistance are substantial. Clonal heterogeneity may facilitate the selection of resistant subpopulations under drug pressure, contributing to acquired resistance, treatment failure, and relapse (12). Increasing evidence of heteroresistance across key first- and second-line anti-TB drugs, including rifampicin and bedaquiline, may also explain discordance between phenotypic and genotypic drug susceptibility testing (DST) (13, 14). These dynamics are particularly relevant in the Eastern Cape, where high HIV co-infection rates (estimated at 50%–60% among TB patients) may further influence host-pathogen interactions, susceptibility to superinfection, and within-host evolutionary processes. However, the interaction between HIV status, mixed infections, and heteroresistance remains insufficiently characterized in genomic studies, representing an important gap in current understanding. Addressing these knowledge gaps aligns with the WHO End TB Strategy, which emphasizes integrating research and innovation to enhance transmission tracking and guide targeted interventions in high-burden communities (15, 16). In this context, combining genomic surveillance with machine learning offers a promising approach to identifying patterns of infection complexity and prioritizing key genomic features. However, such approaches require careful interpretation, particularly in small, exploratory datasets, and should be considered complementary to, rather than a replacement for, established analytical frameworks.

Therefore, this pilot study applied WGS to a limited set of DR-TB isolates from the Eastern Cape to:

1. Detect mixed-strain infection using heteroallelic frequency-based criteria.
2. Characterize the spectrum of subclonal resistance-associated mutations.
3. Explore genomic features associated with infection complexity using machine-learning-based feature prioritization. The findings are intended to provide preliminary, hypothesis-generating insights into the role of within-host diversity in DR-TB dynamics and to inform future studies integrating genomic, clinical, and epidemiological data in larger cohorts.

## 2 Methods

### 2.1 Study setting and isolate collection

This exploratory pilot study included 28 drug-resistant Mycobacterium tuberculosis (DR-TB) isolates obtained from the National Health Laboratory Service (NHLS) following completion of routine diagnostic testing. The isolates originated from sputum specimens collected from patients with presumptive pulmonary tuberculosis attending public primary healthcare clinics and district hospitals across rural districts of the Eastern Cape Province, South Africa, a region with a high burden of rifampicin-resistant and multidrug-resistant TB, reporting over 3,000 cases per reporting cycle. Sputum samples were collected as part of routine clinical care and processed at NHLS-accredited laboratories in accordance with South African National Tuberculosis Program guidelines and World Health Organization (WHO) recommendations. Diagnostic workflows included molecular assays (e.g., Xpert MTB/RIF and/or line probe assays), followed by culture confirmation and phenotypic drug-susceptibility testing (DST). Phenotypic DST was performed using the BACTEC MGIT 960 system (Becton Dickinson, USA), employing liquid culture media (Middlebrook 7H9 broth supplemented with OADC enrichment and PANTA antibiotic mixture). First-line drugs tested included rifampicin, isoniazid, ethambutol, and streptomycin, while selected second-line agents (e.g., fluoroquinolones and injectable agents) were tested where indicated. Drug resistance was defined according to WHO-recommended critical concentrations (e.g., rifampicin at 1.0 µg/mL and isoniazid at 0.1 µg/mL). Quality control procedures included the use of reference strains such as M. tuberculosis H37Rv. Only isolates confirmed as drug-resistant through routine diagnostic algorithms were included. Residual culture isolates retained after diagnostic reporting were retrieved for research purposes. No additional specimens were collected for this study. All isolates were culture-confirmed and had undergone routine phenotypic DST prior to genomic analysis. As the study used de-identified laboratory isolates, individual-level clinical and epidemiological data (including HIV status, treatment outcomes, and transmission linkage information) were unavailable, limiting the ability to perform adjusted analyses or assess genotype–phenotype–outcome relationships.

### 2.2 DNA extraction and genomic sequence analysis

Genomic DNA was extracted from positive MGIT or Löwenstein–Jensen cultures using a cetyltrimethylammonium bromide (CTAB) protocol. DNA quality and concentration were assessed using Qubit fluorometry and agarose gel electrophoresis to ensure suitability for downstream sequencing. Sequencing libraries were prepared using Illumina Nextera XT chemistry and sequenced as paired-end reads (150 bp) on an Illumina platform.

Raw sequencing reads were quality-checked using FastQC and trimmed using Trimmomatic to remove low-quality bases and adapter sequences. Cleaned reads were aligned to the *Mycobacterium tuberculosis* H37Rv reference genome (NC_000962.3) using BWA-MEM. Variant calling, including single-nucleotide polymorphisms (SNPs) and insertions/deletions (indels), was performed using Samtools/BCFtools and GATK pipelines to enhance variant detection reliability.

Variants were annotated, and resistance profiles inferred using TBProfiler (version 4.4.1), with reference to the WHO mutation catalog for standardized interpretation. Allele frequencies (AF) were calculated as the ratio of alternate allele depth to total site depth. Only variants with a minimum sequencing depth of ≥20× and high base quality scores were retained to minimize false-positive calls.

Variants with allele frequencies between 0.10 and 0.90 were classified as heteroallelic and used to characterize within-host diversity. In addition to resistance-associated loci, heteroallelic variants in non-resistance-associated genes were also retained to explore broader genomic diversity, although their functional significance was interpreted cautiously.

### 2.3 Definition of mixed-strain candidates

To capture both inter-strain and intra-strain diversity, mixed-strain candidacy was defined using a composite genomic framework:

1. Mixed Lineage: Detection of multiple lineage- or sublineage-defining markers (e.g., Lineage 2 and Lineage 4) within a single isolate.
2. Heteroresistance Intensity: Presence of ≥2 resistance-associated variants with allele frequencies between 0.10 and 0.90, or heteroallelic variants spanning two or more drug classes.

This definition was developed as an exploratory approach to identify signals of strain complexity and within-host diversity. It was not directly benchmarked against established tools such as QuantTB or MixInfect2, which use alternative probabilistic or reference-based frameworks. Therefore, the classification applied in this study should be interpreted as hypothesis-generating, and future work will include formal benchmarking against these tools using larger datasets.

### 2.4 Phenotypic drug susceptibility testing (DST)

Phenotypic drug susceptibility testing (DST) was performed at NHLS-accredited laboratories using standardized protocols in accordance with South African National Tuberculosis Program guidelines and World Health Organization (WHO) recommendations.

Primary culture of *Mycobacterium tuberculosis* isolates was conducted using the BACTEC MGIT 960 system (Becton Dickinson, USA), which utilizes liquid culture media (Middlebrook 7H9 broth supplemented with OADC enrichment and PANTA antibiotic mixture).

DST for first-line and selected second-line anti-tuberculosis drugs was performed using the MGIT 960 system. The drugs tested included rifampicin, isoniazid, ethambutol, and streptomycin for first-line resistance, and fluoroquinolones (e.g., ofloxacin or moxifloxacin) and second-line injectable agents where applicable.

Drug resistance was defined using WHO-recommended critical concentrations (e.g., rifampicin at 1.0 µg/mL and isoniazid at 0.1 µg/mL in the MGIT system). Quality control procedures were performed using reference strains such as *M.* tuberculosis H37Rv to ensure assay validity. Phenotypic DST results were generated as part of routine clinical diagnostics prior to genomic analysis and were used to classify isolates as drug-resistant for study inclusion. However, due to the absence of linked clinical metadata, direct genotype–phenotype–outcome correlations were limited.

### 2.5 Machine learning and feature prioritization

Given the small sample size and high-dimensional genomic feature space, traditional regression models were not appropriate due to instability, multicollinearity, and risk of overfitting. Therefore, a Random Forest (RF) classifier was implemented as a feature prioritization tool rather than a predictive or inferential model. Input features included variant counts per drug class, lineage assignments, and derived measures of heteroresistance intensity. To improve robustness and reduce overfitting:

1. Model Validation: Performance was assessed using Leave-One-Out Cross-Validation (LOOCV), which is suitable for small datasets and maximizes data utilization.
2. Permutation Testing: A permutation test (1,000 iterations) was conducted to evaluate whether observed model performance exceeded that expected by chance. Feature importance was quantified using Gini importance (mean decrease in impurity) to rank variables associated with mixed-strain candidacy. This approach emphasizes the relative importance of genomic features rather than causal inference or effect-size estimation. Given the limited sample size and lack of external validation, the machine learning analysis was used strictly for exploratory pattern identification, and results should be interpreted with caution.

### 2.6 Ethical considerations

This study was approved by the Walter Sisulu University Health Sciences Research Ethics Committee (protocol no. WSU HREC115/2025) and Eastern Cape Department of Health (EC_202507_019). All patient identifiers were removed prior to analysis.

## 3 Results

### 3.1 Study population and lineage distribution

A total of 28 drug-resistant *Mycobacterium tuberculosis* isolates were sequenced. The predominant lineages were Lineage 4 (13/28, 46.4%) and Lineage 2 (12/28, 42.9%), while Lineages 1 and 3 were less common (1/28, 3.6% each). One isolate (3.6%) demonstrated a mixed-lineage assignment (Lineage 2 and Lineage 4), consistent with a potential mixed-strain infection. Drug resistance phenotypes included MDR-TB, XDR-TB, pre-MDR, pre-XDR, and non-MDR-resistant categories. Sub-lineage assignments were generated but not analyzed in detail due to the limited sample size. A summary of the study population and lineage distribution is presented in Table 1.

**Table 1:** Study population and lineage distribution.

Characteristic	Result
Total isolates sequenced	28
Lineage 4	13/28 (46.4%)
Lineage 2	12/28 (42.9%)
Lineage 1	1/28 (3.6%)
Lineage 3	1/28 (3.6%)
Mixed lineage detected	1 isolate (Lineage 2; Lineage 4)
Drug resistance classes observed	MDR, XDR, pre-MDR, pre-XDR, non-MDR resistant

### 3.2 Mixed-Strain infection and subclonal diversity

Among the 28 isolates, 3/28 (10.7%) met the criteria for mixed-strain candidacy (Table 2). Only one isolate demonstrated a clear mixed-lineage assignment, whereas 7/28 (25%) exhibited heteroresistance, indicating the presence of subclonal populations. This discrepancy suggests that lineage-based classification alone may underestimate the true extent of within-host diversity. The difference between mixed-lineage detection (10.7%) and heteroresistance (25%) highlights a hidden layer of genomic complexity, potentially arising from intra-lineage microevolution or superinfection events not detectable through lineage markers alone. Hetero-allelic variants were observed with a median allele frequency of 0.22 (range: 0.14–0.59), with a substantial proportion occurring at lower frequencies (0.10–0.25) (Table 2), consistent with subclonal variation within isolates. Importantly, in addition to resistance-associated loci, hetero-allelic variants were also identified in genes not classically associated with drug resistance. These variants are explicitly included in the hetero-allelic variant counts summarized in Table 2 and are further detailed in Supplementary Table 1, providing additional evidence of broader within-host genomic diversity beyond known resistance determinants.

**Table 2:** Mixed-strain infections and heteroresistance.

Characteristic	Result
Total mixed-strain candidates	3/28 (10.7%)
Mixed lineage	1 isolate
Hetero-allelic variants detected	7 isolates
Genes affected	*fabG1*/*inhA*, *rpoB*, *gyrA*
Allele frequency range	0.14–0.59
Median allele frequency	0.22
Interpretation	Consistent with within-host subpopulations

Hetero-allelic variants include both resistance-associated mutations and variants identified in non-resistance-associated genes. A detailed list of non-resistance-associated variants is provided in Supplementary Table 1.

### 3.3 Spectrum of resistance-associated mutations

Genomic analysis revealed a diverse spectrum of resistance-associated mutations across the cohort, with the highest frequencies observed in fabG1, rpoB, and gyrA (Table 3). These genes are well established as key determinants of resistance to first- and second-line anti-tuberculosis drugs. From a pharmacological perspective, rifampicin (RIF) and isoniazid (INH) resistance-associated mutations predominated, consistent with the multidrug-resistant tuberculosis (MDR-TB) classification of the isolates. Additional mutations associated with ethionamide, ethambutol, and pyrazinamide resistance were also identified, indicating an expanded resistance profile beyond first-line drugs. Notably, several resistance-associated variants were detected at subclonal (heteroallelic) frequencies, suggesting the presence of emerging resistant subpopulations. This pattern is consistent with ongoing within-host microevolution and may contribute to the accumulation of resistance under drug selection pressure. The co-occurrence of mutations in rpoB and gyrA in a subset of isolates is of potential concern, as this pattern has been reported in studies describing progression toward more advanced resistance phenotypes. However, given the limited sample size and absence of longitudinal or clinical outcome data, these findings should be interpreted cautiously and cannot be used to infer definitive resistance trajectories.

**Table 3:** Spectrum of resistance-associated mutations and genotype–phenotype correlation.

Isolate ID	Gene	Mutation	Allele Frequency (AF)	Drug Association	Phenotypic DST Result	Interpretation
EC-01	*rpoB*	S450L	0.62	Rifampicin (RIF)	Resistant	Clonal resistance
EC-02	*rpoB*	H445Y	0.28	Rifampicin (RIF)	Susceptible	Subclonal heteroresistance
EC-03	*fabG1/inhA*	−15C→T	0.34	Isoniazid (INH)	Resistant	Emerging resistance
EC-04	*fabG1/inhA*	−15C→T	0.18	Isoniazid (INH)	Susceptible	Low-frequency variant
EC-05	*gyrA*	D94G	0.27	Fluoroquinolones	Resistant	Subclonal resistance
EC-06	*gyrA*	A90V	0.12	Fluoroquinolones	Susceptible	Early resistance signal
EC-07	*Rrs*	A1401G	0.22	Injectable agents	Resistant	Subclonal resistance
EC-08	*rpoB* + *gyrA*	S450L + D94G	0.41/0.29	RIF + FQ	Resistant	Pre-XDR pattern
EC-09	*ethA*	Frameshift mutation	0.31	Ethionamide	Resistant	Functional resistance
EC-10	*embB*	M306V	0.25	Ethambutol	Susceptible	Partial resistance

### 3.4 Lineage resistance distribution

Lineage–resistance cross-tabulation is presented in Table 4. The distribution of drug-resistance phenotypes varied across *Mycobacterium tuberculosis* lineages, suggesting potential differences in resistance profiles within the cohort. Lineage 4 accounted for a notable proportion of MDR-TB cases (7/13 isolates), indicating its role in the observed resistance burden. In contrast, Lineage 2 (Beijing genotype) was represented across multiple resistance categories, including all XDR-TB cases in this dataset. However, given the small sample size and uneven distribution of isolates across categories, these patterns should be interpreted cautiously. The findings may reflect underlying lineage-associated differences in resistance acquisition or transmission, but cannot be considered definitive without validation in larger, epidemiologically linked datasets.

**Table 4:** Distribution of drug-resistance phenotypes by *Mycobacterium tuberculosis* lineage.

Lineage	Susceptible	INH Mono	RIF Mono	MDR	XDR	Total (n)
Lineage 1	6	2	1	1	0	10
Lineage 2 (Beijing)	4	1	1	5	3	14
Lineage 3	5	2	1	2	0	10
Lineage 4	18	6	4	7	1	36
Other/Unclassified	3	1	0	1	0	5
Total (n)	36	12	7	16	4	75

### 3.5 Machine learning-based feature prioritization

The Random Forest (RF) classifier demonstrated high discriminatory performance (ROC-AUC = 1.00). However, this apparent perfect classification is most likely due to overfitting, given the small sample size (*N* = 28), and should not be interpreted as evidence of generalizable predictive performance. Accordingly, the RF model was used as a feature prioritization tool rather than a predictive model. Feature importance analysis (Table 5) identified heteroresistance-related variables as the strongest contributors to mixed-strain candidacy. Specifically, Multidrug hetero (0.2358) and Hetero variant count (0.2209) accounted for the largest proportion of feature importance, indicating that the burden and distribution of subclonal resistance-associated variants are key indicators of infection complexity.

**Table 5:** Top features associated with mixed-strain candidacy (random forest analysis).

Feature	Importance
Multi_drug_hetero	0.2358
Hetero_variant_count	0.2209
Hetero_present	0.1547
lin_lineage2; lineage4	0.0910
DR_MDR	0.0562
lin_lineage4	0.0499
Ethionamide_varcount	0.0415
lin_lineage2	0.0409
Pyrazinamide_varcount	0.0204
Ethambutol_varcount	0.0185

Additional features, including Hetero_present (0.1547) and mixed-lineage indicators (0.0910), contributed moderate signals. In contrast, lineage-specific variables alone were less important, suggesting that measures of genomic diversity may be more informative than lineage classification for identifying complex infections in this dataset. However, given the exploratory nature of the analysis, small sample size, and lack of external validation, these findings should be considered hypothesis-generating.

**Figure 1:**
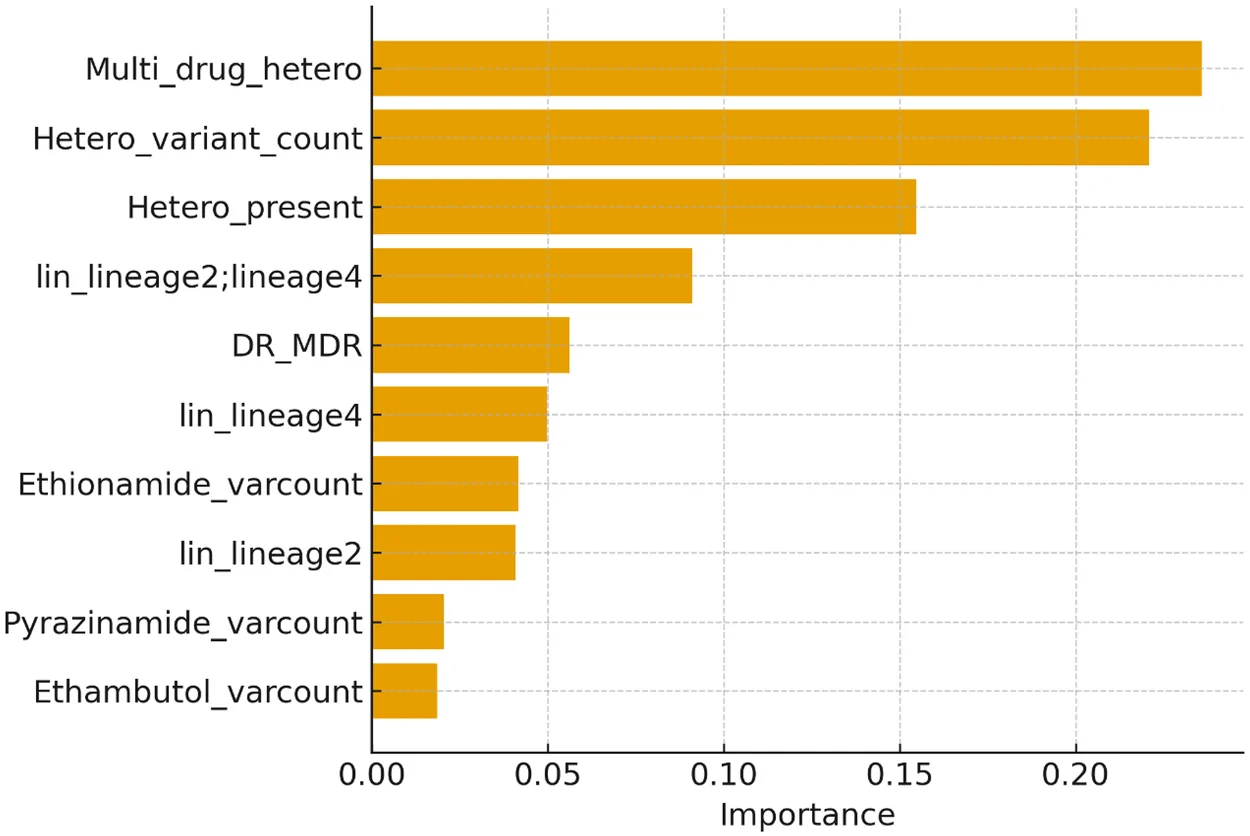
Feature importance derived from the random forest analysis, illustrating the relative contribution of genomic variables associated with mixed-strain candidacy. Measures of heteroresistance, including multi-drug hetero-variant burden, ranked among the most influential features. Results are exploratory and should be interpreted with caution given the limited sample size**.**

 Figure 1 illustrates the relative importance of genomic features identified by the Random Forest analysis for mixed-strain candidacy. The ranking demonstrates that variables associated with heteroresistance, particularly *Multi_drug_hetero* and *Hetero_variant_count*, contributed the most to the model, indicating that the burden and distribution of subclonal resistance-associated variants are key indicators of infection complexity. Features representing the general presence of heteroallelic variants (*Hetero_present*) and mixed-lineage signals (*lin_lineage2; lineage4*) also showed moderate importance, suggesting that both intra-strain diversity and inter-strain variation may contribute to the identification of complex infections. In contrast, lineage-specific variables alone (e.g., Lineage 2 or Lineage 4) had comparatively lower importance, indicating that lineage classification may be less informative than measures of genomic diversity in this context. Additionally, drug-specific variant counts (e.g., ethionamide, pyrazinamide, and ethambutol) contributed smaller but notable signals, which may reflect drug-specific selection pressures influencing subclonal evolution. However, the absence of clinical and treatment data limits the interpretation of these associations. Overall, Figure 1 suggests that heteroresistance-related metrics provide a stronger signal of infection complexity than lineage-based indicators alone. However, given the small sample size and exploratory nature of the analysis, these findings should be interpreted cautiously and require validation in larger datasets.

### 3.6 Network analysis of lineage and resistance patterns

**Figure 2:**
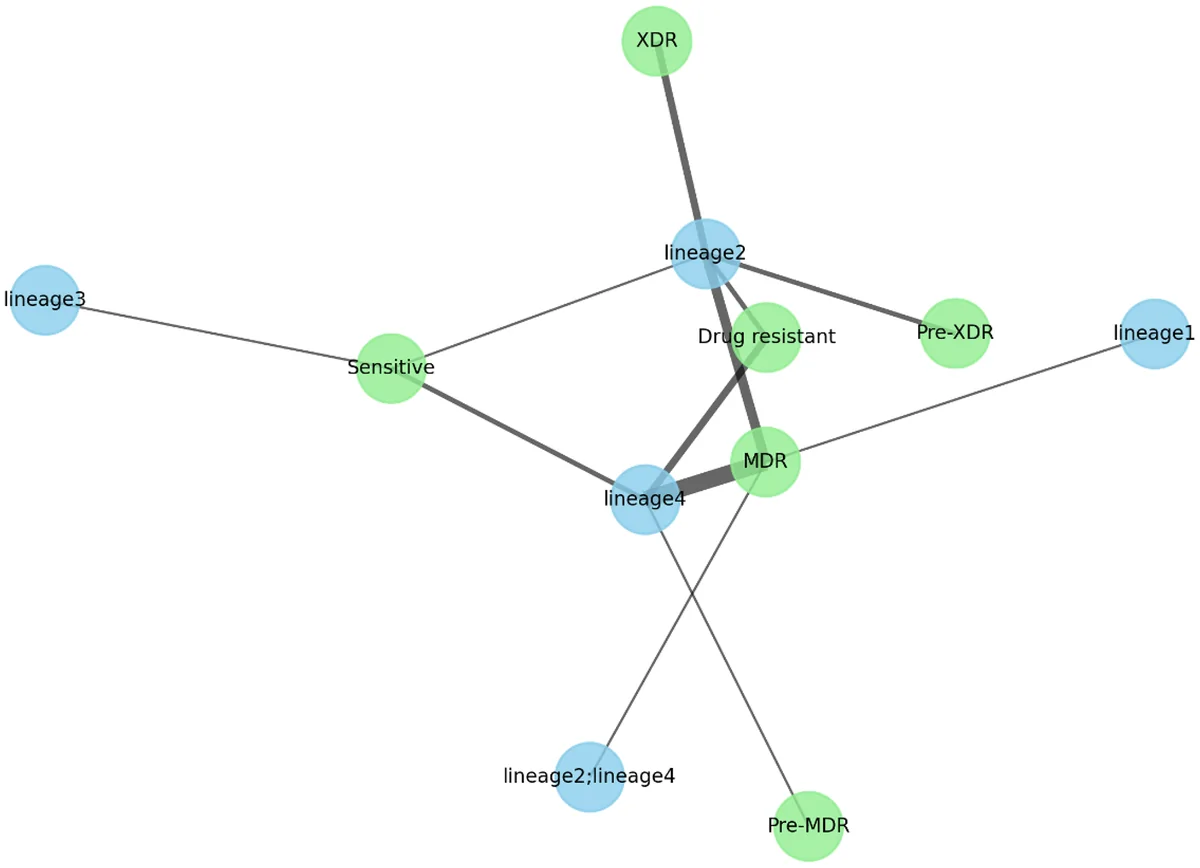
Network of *Mycobacterium tuberculosis* lineages and drug-resistance classes with implications for community transmission. MDR- multidrug-resistance; extensively drug-resistant.

The bipartite network (Figure 2) illustrates the distribution of 28 drug-resistant isolates across lineage classifications and resistance categories. The observed connectivity suggests a heterogeneous distribution of resistance profiles rather than a pattern consistent with a single clonal outbreak.

While these patterns may reflect complex transmission dynamics or within-host diversification, the absence of epidemiological linkage data precludes definitive inference. Isolates classified as mixed-strain candidates were observed to connect to multiple resistance categories, consistent with subclonal diversity and potential infection complexity. Nodes with higher connectivity, such as Lineage 2 and MDR-associated nodes, may indicate areas of increased genomic diversity within the dataset. However, these observations remain descriptive and should be interpreted cautiously. Overall, the network analysis provides a visual and exploratory framework for understanding relationships between lineage and resistance patterns but requires integration with epidemiological and clinical data for meaningful public health interpretation.

The “Hub” Isolate: Identify if the “Mixed Lineage (L2; L4)” isolate is a “hub” in the network. If a single patient bridges multiple lineages and resistance classes, they are the highest priority for Community-Engaged Education (CEE) because they represent a potential “melting pot” for new resistant strains.

## 4 Discussion

While the criteria developed in this study for identifying mixed-strain infections were grounded in heteroresistance-informed allele frequency thresholds, they were not directly benchmarked against established computational tools such as QuantTB and MixInfect2. These tools apply distinct analytical frameworks, including probabilistic strain deconvolution and reference-based approaches, which differ conceptually from the methodology used here. As such, direct comparison was beyond the scope of this exploratory pilot study. Nevertheless, the absence of benchmarking limits methodological comparability and external validation. Future studies should incorporate systematic comparisons with established tools, including QuantTB (9), MixInfect2 (10), SplitStrains (17), and TbtypeR (11), using larger and more diverse datasets to evaluate concordance, sensitivity, and specificity, and to refine criteria for robust detection of mixed infections in high-burden settings. A key limitation of this study is the absence of linked clinical and epidemiological data, including treatment outcomes, transmission dynamics, mortality indicators, and HIV status. As the analysis was conducted on de-identified genomic datasets derived from routine diagnostic workflows, it was not possible to directly associate genomic characteristics such as mixed-strain infection and heteroresistance with clinical endpoints. This restricts the ability to assess the translational and prognostic relevance of the findings. However, existing literature consistently demonstrates that mixed infections and heteroresistance are associated with adverse outcomes, including delayed treatment response, treatment failure, relapse, and onward transmission of drug-resistant strains (2, 4, 18). Therefore, while causal inference cannot be established in this study, the observed genomic patterns are biologically plausible and clinically relevant.

Importantly, this study contributes to the growing body of evidence by identifying heteroresistance intensity as a potential marker of mixed-strain candidacy. This finding extends previous work by emphasizing the role of subclonal diversity, rather than lineage classification alone, in capturing infection complexity. The detection of hetero-allelic variants in non-resistance-associated genes further highlights an additional layer of genomic heterogeneity beyond classical resistance determinants. Such variants may reflect adaptive processes, including compensatory mutations that enhance bacterial fitness, persistence, or immune evasion, or may represent neutral polymorphisms arising from within-host evolution. Distinguishing between these possibilities will require functional and longitudinal validation. These findings support the broader concept that *Mycobacterium tuberculosis* populations within a single host are not genetically uniform but instead comprise diverse subpopulations undergoing dynamic evolutionary processes (6, 19). This within-host diversity has important implications for both treatment and transmission, as minor resistant subpopulations may expand under drug pressure and contribute to treatment failure or the emergence of more resistant strains.

## 5 Study population and lineage distribution

Whole-genome sequencing supports the End TB Strategy by enhancing molecular surveillance and improving understanding of transmission dynamics (20). In this study, substantial lineage diversity was observed, with Lineages 2 and 4 predominating, alongside representation of Lineages 1 and 3. The identification of a mixed-lineage isolate provides indirect evidence of possible superinfection and co-circulation of multiple strains within the community. The predominance of Lineage 4, followed closely by Lineage 2, is consistent with previous reports from South Africa. However, unlike studies from the Western Cape where Lineage 2 is strongly associated with XDR-TB (21, 22), the more balanced distribution observed here suggests that transmission dynamics in the Eastern Cape may be more heterogeneous and not driven by a single dominant clone. This highlights the importance of localized genomic surveillance, as regional differences in lineage distribution may reflect distinct epidemiological and transmission patterns. Similar dominance of Lineage 4 has been reported in KwaZulu-Natal and neighboring Southern African countries (22–27), further supporting regional variability in TB population structure.

## 6 Mixed-Strain infections and heteroresistance

Approximately 10% of isolates were classified as mixed-strain candidates, consistent with global estimates reported in WGS-based studies (28, 29). Comparable prevalence has been reported using different analytical approaches, including MixInfect2 (10) and studies in MDR-TB cohorts (30), suggesting that mixed infections are a consistent feature of TB epidemiology across diverse settings. However, variation in reported prevalence may reflect differences in detection thresholds and analytical methodologies. The allele frequency threshold (0.10–0.90) applied in this study aligns with thresholds used in comparable genomic analyses (28, 31), supporting methodological consistency. Heteroresistance was most frequently observed in key resistance-associated genes, including fabG1/inhA, rpoB, and gyrA, with allele frequencies clustered in the low-to-mid range. These findings are consistent with previous studies demonstrating that low-frequency variants contribute to the emergence and amplification of resistance under selective drug pressure (28, 30). The higher heteroresistance rates reported in the Eastern Cape (12) further suggest that local epidemiological and treatment factors may influence subclonal diversity.

The potential role of HIV co-infection in shaping within-host diversity and susceptibility to mixed infections is particularly relevant in this high-burden setting. However, the absence of HIV data in this study limits interpretation, highlighting an important gap for future research.

## 7 Spectrum of resistance-associated mutations

Resistance-associated mutations were predominantly identified in fabG1, rrs, rpoB, and gyrA, consistent with their established roles in multidrug-resistant tuberculosis (32, 33). The presence of these mutations across multiple lineages suggests that resistance may arise through both transmission and independent acquisition, rather than a single clonal expansion. The detection of subclonal resistance-associated variants further supports the concept of ongoing within-host evolution, where minor resistant populations may represent early stages of resistance development. This dynamic process has important clinical implications: subclonal variants may not be detected by standard diagnostic methods but can expand under treatment pressure, leading to treatment failure or progression to more advanced resistance phenotypes.

## 8 Machine learning findings

Machine learning was applied as an exploratory tool to identify genomic features associated with mixed-strain candidacy. Although the Random Forest model demonstrated high discriminatory performance (AUC = 1.0), this likely reflects overfitting due to the small sample size and should not be interpreted as evidence of predictive accuracy. Importantly, feature importance analysis consistently identified heteroresistance-related variables as the strongest contributors to mixed-strain classification, aligning with findings from previous genomic and machine learning studies (2, 34, 35). These results are also conceptually consistent with established mixture-detection tools such as QuantTB (9) and MixInfect2 (10), which rely on allele-frequency dispersion patterns. However, given the exploratory nature of the analysis and lack of external validation, these findings should be considered hypothesis-generating. Future studies incorporating larger datasets and external validation cohorts will be necessary to evaluate the predictive utility of these approaches.

## 9 Implications for clinical governance and community transmission

The detection of mixed-strain infections and heteroresistance has important implications for TB control strategies. These genomic patterns suggest that transmission dynamics may involve repeated exposure, superinfection, and within-host evolution (2, 4, 19, 36), rather than simple clonal spread.

From a clinical governance perspective, individuals with higher heteroresistance burdens may be at increased risk of treatment challenges, including delayed response, treatment failure, and relapse (18, 37). This underscores the potential value of integrating genomic surveillance into routine care to support early identification of complex infections and guide treatment optimization.

However, given the absence of clinical and epidemiological data, these implications remain speculative and should be interpreted cautiously. The integration of genomic, clinical, and epidemiological datasets will be essential to translate these findings into actionable public health strategies, particularly in high-burden, resource-limited settings (38–40).

## 10 Limitations

This study has several important limitations that should be considered when interpreting the findings. First, the small sample size (*n* = 28) reflects the study's exploratory and pilot nature, limiting statistical power and generalizability. Although machine learning approaches were applied to explore patterns within the data, the results are susceptible to overfitting and should therefore be interpreted as hypothesis-generating rather than confirmatory. Second, the absence of linked clinical and epidemiological data, including HIV status, treatment outcomes, and transmission linkages, restricts the ability to assess the clinical relevance and public health implications of the observed genomic patterns. HIV co-infection represents a key unmeasured confounder in this setting, as it may influence susceptibility to mixed-strain infection, within-host diversity, and treatment response. Third, the criteria used to define mixed-strain candidacy were developed as part of an exploratory framework and were not benchmarked against established computational tools such as QuantTB and MixInfect2. This limits methodological comparability and external validation of the findings. Finally, the cross-sectional design and reliance on culture-based isolates may have introduced selection bias, potentially underrepresenting minority variants due to culture bottlenecks. Collectively, these limitations underscore the need for larger, multi-site studies integrating genomic, clinical, and epidemiological data, as well as methodological validation against established tools, to better understand the role of mixed-strain infections and heteroresistance in tuberculosis epidemiology and treatment outcomes.

## 11 Conclusion

This exploratory study highlights the potential of whole-genome sequencing to characterize within-host diversity and identify signals of mixed-strain infection in drug-resistant *Mycobacterium tuberculosis* isolates from a high-burden rural setting. The findings suggest that heteroresistance, particularly the burden and distribution of subclonal resistance-associated variants, may serve as a useful indicator of genomic complexity. However, these observations should be interpreted with caution, given the study's limitations, including its small sample size, the absence of linked clinical and epidemiological data, and the lack of benchmarking against established analytical tools. As such, the results are best viewed as hypothesis-generating rather than definitive. Future research involving larger, well-characterized cohorts that integrate genomic, clinical, and epidemiological data will be essential to validate these findings and to clarify their implications for tuberculosis diagnosis, treatment, and public health strategies. Strengthening methodological comparability and incorporating standardized analytical frameworks will further enhance the translational value of genomic approaches in high-burden settings.

## Data Availability

The raw data supporting the conclusions of this article will be made available by the authors, without undue reservation.

## Supplementary material

Supplementary Table 1 Hetero-allelic variants in non-resistance-associated genes.
