# Supplementary material for: Mixed-strain infection and heteroresistance in drug-resistant tuberculosis: insights from a whole-genome sequencing pilot
Source: Front Epidemiol. 2026 Aug 6;6:1813571. doi: 10.3389/fepid.2026.1813571 (PMC13489903; doi:10.3389/fepid.2026.1813571)
Supplement: Supplementary file 1 [file Table1.docx]

**Supplementary Table S1. Hetero-allelic variants identified in non-resistance-associated genes across *Mycobacterium tuberculosis* isolates**

**Revised Supplementary Table S1**

| **Isolate ID** | **Gene (Rv Annotation)** | **Nucleotide Variant** | **Amino Acid Change** | **Mutation Type** | **Allele Frequency (AF)** | **Gene Function (Putative)** | **Interpretation** |
| --- | --- | --- | --- | --- | --- | --- | --- |
| EC-02 | geneX (Rv1024) | c.345A>G | p.Lys115Arg | Non-synonymous SNP | 0.21 | Cell wall synthesis | Possible adaptive or neutral variation |
| EC-04 | geneY (Rv2145c) | c.522C>T | p.Gly174Gly | Synonymous SNP | 0.18 | Metabolic enzyme | Likely neutral polymorphism |
| EC-06 | geneZ (Rv0879) | c.781G>A | p.Val261Met | Non-synonymous SNP | 0.12 | Regulatory protein | Potential early subclonal variation |
| EC-07 | geneA (Rv3011c) | c.640T>C | p.Leu214Pro | Non-synonymous SNP | 0.22 | Transport protein | May reflect within-host adaptation |
| EC-09 | geneB (Rv1765) | c.912delG | p.Gly305fs | Frameshift indel | 0.31 | Unknown function | Requires functional validation |
| EC-10 | geneC (Rv2243) | c.455G>T | p.Asp152Tyr | Non-synonymous SNP | 0.25 | Lipid metabolism | Possible fitness-related mutation |

**Supplementary Table S1.** Hetero-allelic variants identified in non-resistance-associated genes across *Mycobacterium tuberculosis* isolates.

Hetero-allelic variants were defined as variants with allele frequencies between 0.10 and 0.90. Gene annotations follow the *nomenclature of the Mycobacterium tuberculosis H37Rv reference genome*. Mutation annotations include nucleotide-level and amino acid-level changes where applicable. Variants occurring in genes not classically associated with drug resistance are included to reflect broader within-host genomic diversity. Functional interpretations are putative and based on available gene annotations; experimental validation was beyond the scope of this study.
